# Executive Functions and Psychopathology Dimensions in Deficit and Non-Deficit Schizophrenia

**DOI:** 10.3390/jcm12051998

**Published:** 2023-03-02

**Authors:** Maksymilian Bielecki, Ernest Tyburski, Piotr Plichta, Monika Mak, Jolanta Kucharska-Mazur, Piotr Podwalski, Katarzyna Rek-Owodziń, Katarzyna Waszczuk, Leszek Sagan, Shane T. Mueller, Anna Michalczyk, Błażej Misiak, Jerzy Samochowiec

**Affiliations:** 1 Department of Health Psychology, Pomeranian Medical University in Szczecin, 71-457 Szczecin, Poland; 2 Department of Psychiatry, Pomeranian Medical University in Szczecin, 71-457 Szczecin, Poland; 3 Department of Neurosurgery, Pomeranian Medical University in Szczecin, 71-252 Szczecin, Poland; 4 Department of Cognitive and Learning Sciences, Michigan Technological University, Houghton, MI 49931, USA; 5 Department of Psychiatry, Wroclaw Medical University, 50-367 Wroclaw, Poland

**Keywords:** schizophrenia, executive functions, cognitive functions, psychopathology, deficit schizophrenia, non-deficit schizophrenia

## Abstract

This study: (a) compared executive functions between deficit (DS) and non-deficit schizophrenia (NDS) patients and healthy controls (HC), controlling premorbid IQ and level of education; (b) compared executive functions in DS and NDS patients, controlling premorbid IQ and psychopathological symptoms; and (c) estimated relationships between clinical factors, psychopathological symptoms, and executive functions using structural equation modelling. Participants were 29 DS patients, 44 NDS patients, and 39 HC. Executive functions were measured with the Mazes Subtest, Spatial Span Subtest, Letter Number Span Test, Color Trail Test, and Berg Card Sorting Test. Psychopathological symptoms were evaluated with the Positive and Negative Syndrome Scale, Brief Negative Symptom Scale, and Self-evaluation of Negative Symptoms. Compared to HC, both clinical groups performed poorer on cognitive flexibility, DS patients on verbal working memory, and NDS patients on planning. DS and NDS patients did not differ in executive functions, except planning, after controlling premorbid IQ and negative psychopathological symptoms. In DS patients, exacerbation had an effect on verbal working memory and cognitive planning; in NDS patients, positive symptoms had an effect on cognitive flexibility. Both DS and NDS patients presented deficits, affecting the former to a greater extent. Nonetheless, clinical variables appeared to significantly affect these deficits.

## 1. Introduction

As a mental illness, schizophrenia was first described by Emil Kraepelin in 1883. Over the years, the approach towards the description of its clinical presentation has evolved to finally assume its current form found across the diagnostic classifications used both in clinical practice and scientific research. Cognitive impairment is one of its central features, observed in all stages of schizophrenia, affecting executive function, memory, working memory, language, attention, and processing speed. Despite the prevalence of cognitive dysfunction in patients with schizophrenia, only limited information is available on the underlying neurobiological mechanisms thereof [1,2,3].

Over the years, several models of schizophrenia have been proposed, from homogeneous to multi-factorial concepts. One such approach was to distinguish two separate types of schizophrenia: deficit schizophrenia (DS) and non-deficit schizophrenia (NDS), based on negative (deficit) symptom severity at onset [4], risk factors [5,6], family history [7], disease course [8,9], response to treatment [10], neuropsychological functioning [11,12], and neurobiological differences [13].

Most studies tend to show greater cognitive impairment in DS patients compared to their NDS counterparts. However, it is still unclear whether there are significant differences in terms of the entire cognitive profile in DS relative to NDS [14]. The available literature suggests that there may be certain subpopulations of schizophrenia patients that manifest significant cognitive deficits compared to others [15,16]. Studies also indicate that patients with DS are slightly more susceptible to cognitive interference and have reduced ability to create concepts as well as impaired non-verbal cognitive flexibility [17]. Previous studies suggest that patients suffering from DS manifest more severe negative symptoms, greater disorganization, and less severe affective symptoms [18]. Research also provides evidence for cognitive inhibition deficits in schizophrenia, but it is not entirely clear whether they are reflected in behavior [19].

According to Carpenter et al. [20], deficit symptoms—primary, persistent negative symptoms, such as withdrawal from social contact, impoverished speech, and apathy or limited affect [21]—dominate the clinical presentation of DS and are present throughout the course of the disease. Longitudinal analyses indicate that they are also stable over time [4]. Executive functions are understood as higher-order processes responsible for controlling and organizing individual cognitive performance [22]. Behavior control, which enables the pursuit of long-term goals and the resisting of short-term impulses, is an important area of executive functioning that may be more disturbed in patients with DS compared to patients with NDS [23]. Of note, various definitions of executive functions are available in the literature. In broad terms, they may be identified as planning, inhibition, cognitive flexibility, and working memory [24]. To take a more detailed approach, we should also consider the abilities to (1) focus on a given task, (2) select an adequate coping strategy, (3) shift attention, (4) change thought processes, (5) solve complex problems, (6) monitor errors, and (7) inhibit actions [25] as further executive processes. “Executive functions” are also a collective name for functions that involve free actions, such as planning, organizing, self-awareness, self-regulation, and initiation of action [26]. Lezak emphasizes the adaptive role of executive functions, such as abstract or creative thinking, the ability to introspect, and other skills that enable achievement of set goals [27], while other contemporary approaches highlight their importance in the process of integrating actions, cognitions, and emotions, thus regulating human behavior [28].

There is evidence that negative and disorganization symptoms of schizophrenia are significantly linked with executive impairment [29], suggesting that a common frontal dysfunction may underlie these two classes of symptoms. Quite remarkably, the available research findings link negative symptoms with verbal fluency but considerably less so with the inhibition of prepotent responses, suggesting a reverse model in terms of disorganization symptoms; this supports the notion that there may exist quite distinct patterns of executive dysfunction present in schizophrenia patients. Studies to date answer only some of the questions related to the cognitive functioning of schizophrenia patients. Hence, a better understanding of the characteristics of executive performance of DS and NDS patient populations could supplement the existing theoretical models. Of note, most available studies did not control for the effects of premorbid intelligence quotient (IQ) and level of education when comparing executive functions between DS and NDS patients and healthy controls (HC) [14]. Moreover, previous studies have yielded inconclusive results, with some suggesting no differences in premorbid IQ between DS and NDS patients [30,31] but others demonstrating lower premorbid IQ and more impaired executive functions in DS patients compared to NDS patients [32,33,34,35]. In addition, previous studies used neuropsychological tests to measure a smaller range of executive functions and did so in a more selective fashion, such as through problem-solving (e.g., the Wisconsin Card Sorting Test), cognitive flexibility (e.g., the Trail Making Test), or cognitive inhibition (e.g., the Stroop Test) [11,14]. What is more, only a few studies have attempted to investigate the differences while controlling for psychopathology. Despite numerous studies on the relationship between psychopathological symptoms and executive functions in schizophrenia, we still know little about this relationship in DS [16]. Interestingly, despite the utility of the five-factor structure of the Positive and Negative Syndrome Scale (PANSS; including positive symptoms, negative symptoms, disorganization, affect, and resistance) [36] in the assessment of symptom severity, this approach seems to have been under-used in previous studies on DS and cognitive functions. Given these limitations, we set out to (1) compare different aspects of executive functions (using a battery of five neuropsychological tests including several different indices) between DS and NDS patients and HC, controlling for premorbid IQ and level of education; (2) compare these cognitive processes in DS and NDS patients, controlling for premorbid IQ and psychopathological symptoms on the PANSS; and (3) estimate the relationships between clinical factors, psychopathological symptoms, and executive performance using complex structural regression models. Based on the available literature, we proposed three hypotheses. Firstly, we assumed there would be differences between all the groups in executive performance after controlling for education and premorbid IQ. We also hypothesized that there would be no differences between the clinical groups in executive performance after controlling for premorbid IQ and psychopathological symptoms as measured with the PANSS. Our last hypothesis was that a complex relationship would emerge between different psychopathological dimensions and executive functions in both clinical groups.

## 2. Materials and Methods

### 2.1. Participants

This study involved 73 patients diagnosed with schizophrenia based on the International Statistical Classification of Diseases and Related Health Problems (ICD-10) [37] diagnostic criteria and a structured questionnaire (Mini-International Neuropsychiatric Interview; MINI) [38], including 29 patients with a diagnosis of DS (based on the criteria proposed by Carpenter et al. [20]), 44 patients with a diagnosis of NDS, and 39 healthy participants (without mental or neurological disorders). Patients from the clinical group were recruited on the basis of cooperation with psychiatrists working at the Department and Clinic of Psychiatry of the Pomeranian Medical University and Mental Health Clinics. Healthy participants were recruited through information provided by employees and students of the Pomeranian Medical University. The inclusion criteria in the clinical group were: a diagnosis of schizophrenia, illness duration of ≥10 years, being aged 30–50 years, and informed consent to participate in the study. The age criteria were chosen because previous studies have yielded inconclusive results concerning the effect of age, illness duration, and brain changes in older patients with schizophrenia [39,40,41,42,43]; we therefore sought to compare DS and NDS patients as a more homogenous groups. The criteria for inclusion in the comparison group were: being aged from 30 to 50 years and giving informed consent to participate in the study. Exclusion criteria were: the presence of mental illnesses (other than schizophrenia), long-term treatment with benzodiazepines, the presence of neurological diseases that may affect cognitive functioning, alcohol dependence, drug or psychoactive substance addiction, diagnosis of a chronic disease (e.g., heart disease, cancer, endocrine and metabolic diseases or rheumatological diseases) that may affect cognitive functioning, and previous head injuries with loss of consciousness. All participants underwent a psychological and psychiatric examination.

All patients gave written consent to participate in the study. The study protocol was approved by the local bioethics committee.

### 2.2. Neuropsychological Assessment

#### 2.2.1. General Intellectual Ability

General intellectual ability as indirect premorbid IQ was assessed with the Vocabulary and Picture Completion measures of the Wechsler Adult Intelligence Scale—Revised, the standardized measurement of adult general intelligence. Some researchers suggest that intellectual functioning can be associated with executive functions and working memory, both in healthy adults and clinical populations [44]. Moreover, both subtests are often used to measure indirect (case-control studies) and direct (longitudinal studies) premorbid IQ in schizophrenia (meta-analysis by Khandaker et al. [45]) and previous studies have shown that both subtests are strongly related to full IQ [46,47] in schizophrenia patients. Based on the recommendations of many authors [48,49,50,51,52], we selected the Vocabulary subtest as a measure of indirect premorbid crystallized IQ and Picture Completion as a measure of indirect premorbid fluid IQ.

#### 2.2.2. Tasks from MCCB

To measure working memory and planning, we used three tasks from the Polish version of the MATRICS Consensus Cognitive Battery (MCCB) [53,54]: the Letter Number Span Test (LNST), the Spatial Span Subtest (SSS), and the Maze Subtest (MS).

We did not use other tasks from the MCCB, such as the Symbol-Coding, Category Fluency, or Continuous Performance tests, which can be useful for measuring executive functions, as these tasks are believed to involve basic cognitive processes like processing speed and visual sustained attention more than the higher-order cognitive processes (like working memory, central executive system, and planning) that we intended to focus on in this study. Moreover, Category Fluency is a tool that involves one’s mental lexicon and semantic memory, not only executive functions. The LNST was used to assess verbal working memory. In this task, the participant must mentally organize a list of letters and numbers that is presented orally by the researcher and then repeat it back. We then analyzed the sum of correct answers to this task. The SSS from the Wechsler Memory Scale was used to assess visuospatial memory. This task requires the participant to memorize the location of a series of blocks indicated by the person conducting the test, forward and backward, respectively. We analyzed scores on the backward version of this task. The MS from the Neuropsychological Assessment Battery was used to assess planning ability. It includes seven mazes of gradually increasing difficulty laid out on a single sheet of paper to be filled-in with a pencil. Time was measured during the task. We measured speed-dependent planning as the sum of points awarded based on the time taken to solve the mazes.

#### 2.2.3. Color Trail Test

The Color Trail Test (CTT) [55] is a neuropsychological test that examines several attention and executive functions, especially perceptual tracking, sustained and divided attention, sequencing, and self-monitoring. Graphomotor skills are also involved. This study used the Polish version of the CTT (time reaction in part 2) [56] to measure cognitive flexibility.

Participants are shown numbered circles printed with a bright pink or yellow background that can be seen by participants with color blindness. In part 1, the respondent uses a pencil to quickly connect circles numbered 1–25 in sequence. For part 2, the respondent quickly connects sequentially numbered circles, but alternates between pink and yellow. The time taken to complete each trial is recorded, along with qualitative performance characteristics indicative of brain dysfunction, such as near misses, prompts, number sequence errors, and color sequence errors. It retains the sensitivity and specificity of the original trail-making test, but replaces the letters with color, making it more appropriate in cross-cultural contexts and for participants with special needs. The validity of the CTT has been documented in various clinical and neuropsychological populations.

#### 2.2.4. Berg Card Sorting Test

Our study used a computerized version of the Berg Card Sorting Test (BCST) with 64 cards [57] from the Psychology Experiment Building Language (PEBL) [58]. In this task, participants have to discover the rule that is currently operating (color, shape, or number) and answer by pressing one of four number keys (1 to 4) based on the feedback (correct or incorrect) displayed on a 15″ computer screen. Before the test, each participant reads the instructions. This task has previously been used to assess executive functions in schizophrenia [59,60]. Based on Polgár et al. [61], we measured two components of executive functions: concept formation using percent of perseverative errors (PPE) and problem-solving using the percent of non-perseverative errors (PNPE).

### 2.3. Clinical Assessment

To measure the severity of psychopathological symptoms in DS and NDS patients, we used the Positive and Negative Syndrome Scale (PANSS) [62,63]. In analysis, we distinguished five psychopathological dimensions: negative, positive, disorganization, resistance, and affect, as recommended by Shafer and Dazzi [36] based on their meta-analysis of 45 factor analyses of PANSS. Moreover, we used the Polish versions of the Brief Negative Symptom Scale (BNSS) [64] and the Self-evaluation of Negative Symptoms (SNS) [65] to describe deficit symptoms. We assessed the severity of schizophrenia and its impact on functioning using the Global Assessment of Functioning (GAF) [66].

### 2.4. Statistical Analysis

Statistical analysis of the results was performed using IBM SPSS 28 and AMOS 8 (IBM Corp., Redmont, VA, USA). Continuous variables were presented as means (*M*) and standard deviations (*SD*). The normality of the distributions were examined with the Shapiro-Wilk test as well as skewness and kurtosis values. We assumed that skewness and kurtosis values from −2 to +2 indicated normal distributions of variables [67]. Age, years of education, and IQ (based on the Vocabulary subtest of the Wechsler Adult Intelligence Scale Revised Fourth Edition; WAIS-R-IV [68]) were normally distributed in all groups; duration of illness, global functioning on the GAF, and chlorpromazine equivalent were all normally distributed in the clinical groups. Exacerbation, psychopathological symptoms on the PANSS (positive symptoms, negative symptoms, disorganization, affect, resistance, and total score), negative symptoms on the BNSS and SNS, premorbid IQ (measured with the Picture Completion subtest from WAIS-R-IV), and scores on all executive function tasks (LNST, SSS, MS, CTT, and BCST) were non-normally distributed. Therefore, prior to the analyses, we logarithmically transformed exacerbation and Box-Cox transformed the other variables to achieve normal distributions [69]. Differences between the two clinical groups were examined with Student’s *t* test (clinical factors and psychopathological symptoms). Differences between three groups in different aspects of executive functions were examined with analysis of covariance (ANCOVA), controlling for the effect of years of education and premorbid IQ (fluid and crystallized). Moreover, to examine differences in different aspects of executive functions between the two clinical groups, we conducted an ANCOVA to control for the effects of premorbid IQ and psychopathological symptoms on the PANSS. Comparisons between groups were performed using the Bonferroni post hoc test (for parametric tests). Cohen’s *d* and η^2^ (continuous variables) and Cramér’s *V* (categorical variables) were used to determine the magnitudes of effect sizes for differences between groups [70]. Finally, in order to assess the relationships between clinical factors, psychopathological symptoms, and different aspects of executive functions in both clinical groups, Pearson’s *r* correlation coefficients were estimated. Furthermore, Structural Equation Modeling (SEM) was used to investigate the impact of clinical factors and psychopathological symptoms on executive functions (multiple regression model). The selected indices were: the chi-square statistic (χ^2^), the root mean square error of approximation (RMSEA), standardized root mean squared residual (SRMR), the goodness-of-fit index (GFI), and the comparative fit index (CFI). RMSEAs of <0.06, 0.08–0.10, and >0.10 were considered to indicate good, adequate, and poor fit, respectively; SRMR < 0.08, GFI and CFI of >0.90 were considered to indicate an acceptable fit [71]. We used a bootstrap maximum-likelihood estimation with 10,000 samples [72]. The alpha criterion level was set at 0.05 in all statistical analyses.

## 3. Results

### 3.1. Demographic and Clinical Characteristics

Demographic and clinical characteristics are presented in Table 1. There were no significant differences in age; however, the groups differed significantly in years of education (*p* = 0.018), sex (*p* = 0.010), premorbid fluid IQ measured by WAIS-R-IV Picture Completion (*p* < 0.001), and premorbid crystallized IQ measured by WAIS-R-IV Vocabulary (*p* < 0.001). Post hoc analyses showed that DS patients had fewer years of education than HC (*p* = 0.029), had lower fluid IQ than NDS and HC (*p* = 0.010 and *p* < 0.001) as well as lower crystallized IQ than NDS and HC (for both: *p* < 0.001), and that there were more males than females in this group. NDS patients also had lower fluid IQ and crystalized IQ than HC (both: *p* < 0.001). After Holm-Bonferroni *p*-value correction, DS patients had greater severity of negative symptoms and total score on the PANSS (*p* < 0.001 and *p* = 0.01), negative symptoms on BNSS (*p* < 0.001), and negative symptoms on SNS (*p* < 0.001) than NDS patients. The clinical groups did not significantly differ in antipsychotic medications, chlorpromazine equivalent, duration of illness, exacerbation, global functioning on the GAF, or other psychopathological symptoms on the PANSS (positive symptoms, disorganization, affect, or resistance).

### 3.2. Differences in Executive Functions

**Figure 1:**
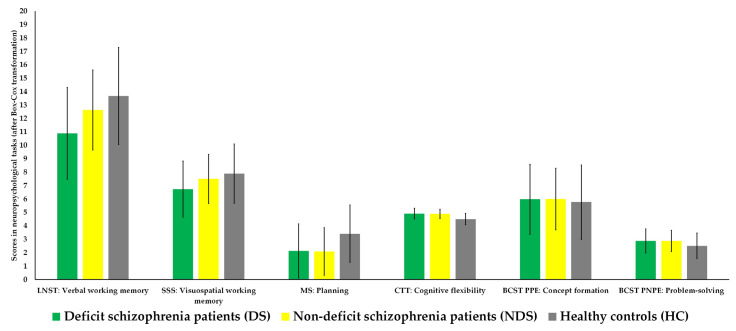
Comparison of executive function profiles between all three groups (scatter bars present SD). BCST = Berg Card Sorting Test; CTT = Color Trail Test; LNST = Letter Number Span Test; MS = Mazes Subtest; SSS = Spatial Span Subtest.

As can be seen in Table 2 and Figure 1, there were significant differences in verbal working memory measured by LNST (*p* = 0.016), planning measured by MS (*p* = 0.017), and cognitive flexibility measured by CTT (*p* < 0.001) between all groups after adjusting for years of education and premorbid fluid and crystallized IQ. Post hoc analysis showed that DS patients had lower scores for verbal working memory (*p* = 0.016) and cognitive flexibility (*p* < 0.001) than did HC. NDS patients had lower scores for planning (*p* = 0.014) and cognitive flexibility (*p* < 0.001) than did HC.

**Figure 2:**
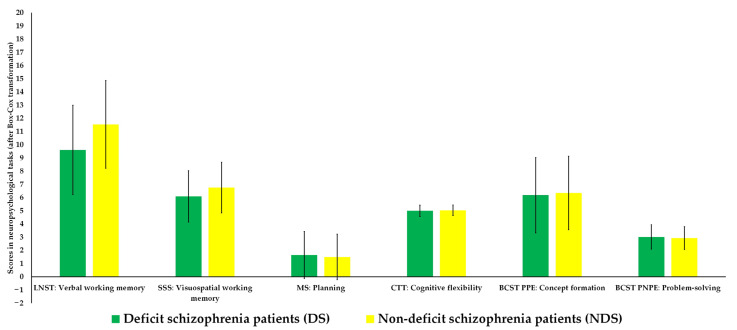
Comparison of executive function profiles between participants from the two clinical groups (scatter bars present SD). BCST = Berg Card Sorting Test; CTT = Color Trail Test; LNST = Letter Number Span Test; MS = Mazes Subtest; SSS = Spatial Span Subtest.

As presented in Table 3 and Figure 2, an additional analysis showed that there were no significant differences in any aspect of executive functions, except planning (*p* = 0.025), between DS and NDS patients after adjusting for premorbid fluid and crystallized IQ, and negative psychopathological symptoms on the PANSS, negative symptoms on BNSS, and negative symptoms on SNS.

### 3.3. Relationships between Psychopathological Dimensions and Executive Functions

As can be seen in Table 4, in DS patients exacerbation correlated negatively with verbal working memory (*r* = −0.47; *p* = 0.010) and planning (*r* = −0.46; *p* = 0.012), and correlated positively with cognitive flexibility (*r* = 0.48; *p* = 0.008). Moreover, global functioning on the GAF correlated positively with planning (*r* = 0.37; *p* = 0.047), while resistance on the PANSS correlated positively with cognitive flexibility (*r* = 0.40; *p* = 0.031). Correlation coefficients have not been corrected because we analyzed many clinical factors and different psychopathological dimensions as predictors of executive functions.

**Figure 3:**
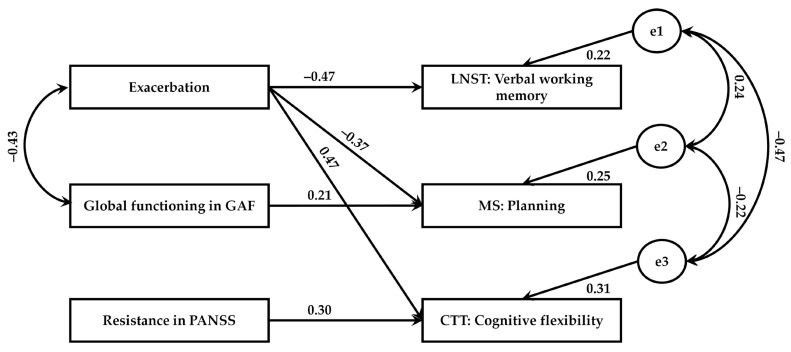
Clinical factors and psychopathological symptoms as predictors of executive functions in DS patients. e1–e3 depict the error term for the dependent variables.

To test the effect of clinical factors and psychopathological symptoms on different aspects of executive functions in this group, we adopted a path analysis methodology within a structural equation modeling (SEM) framework. Considering only significant correlations, we then decided to add selected paths between exacerbation, global functioning on GAF, resistance on PANSS, verbal working memory, planning, and cognitive flexibility to the model. Based on the criteria recommended by Hu and Bentler [46], the model showed good fit to data (χ^2^ = 2.44 and *p* = 0.876; RMSEA = 0.000 and *p* = 0.893; SRMR = 0.070; GFI = 0.973; CFI = 1.000; see Figure 3).

Table 5 shows standardized regression weights for the effects of the clinical factors and psychopathological symptoms on different aspects of executive functions in this group. As shown, exacerbation had an overall effect on verbal working memory (β = −0.472; *p* = 0.003) and cognitive planning (β = 0.472; *p* = 0.005). The recorded values of predicted variance were 22% for verbal working memory and 31% for cognitive flexibility. In general, exacerbation was an important predictor of executive functions. That is, patients who scored higher on exacerbation were likely to have worse verbal working memory and cognitive flexibility.

As can be seen in Table 4, in NDS patients chlorpromazine equivalent correlated negatively with planning (*r* = −0.34; *p* = 0.024), disorganization correlated negatively with planning (*r* = −0.35; *p* = 0.021), and positive symptoms correlated positively with cognitive flexibility (*r* = 0.35; *p* = 0.018). Correlation coefficients have not been corrected because we analyzed many clinical factors and different psychopathological dimensions as predictors of executive functions.

**Figure 4:**
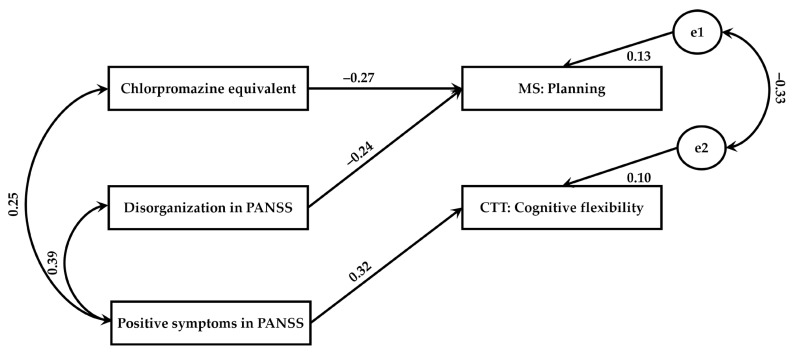
Clinical factors and psychopathological symptoms as predictors of executive functions in NDS patients; e1–e2 depict the error term referring to the dependent variables.

To test the effect of clinical factors and psychopathological symptoms on different aspects of executive functions in this group, we adopted a path analysis methodology within a SEM framework. Considering only significant correlations, we then decided to add selected paths between chlorpromazine equivalent, disorganization, positive symptoms on PANSS, and planning and cognitive flexibility to the model. Based on the criteria recommended by Hu and Bentler [46], the model showed good fit to data (χ^2^ = 5.19 and *p* = 0.269; RMSEA = 0.083 and *p* = 0.322; SRMR = 0.106; GFI = 0.954; CFI = 0.956; see: Figure 4).

Table 6 shows standardized regression weights for the effects of the clinical factors and psychopathological symptoms on different aspects of executive functions in this group. As shown, positive symptoms had an overall effect on cognitive flexibility (β = 0.322; *p* = 0.027). The predicted variance was 10% for cognitive flexibility. In general, positive symptoms were an important predictor of executive functions. That is, patients who scored higher on positive symptoms were likely to have worse cognitive flexibility.

## 4. Discussion

The aim of this study was to determine the differences in executive functions in deficit schizophrenia (DS) and nondeficit schizophrenia (NDS) patients relative to healthy controls (HC) after adjusting for years of education and premorbid IQ. The analysis showed that DS patients had lower scores for verbal working memory and cognitive flexibility than did HC. Patients with NDS scored lower than HC in terms of planning and cognitive flexibility. The DS and NDS groups did not differ significantly from each other. The second objective of the study was to compare DS and NDS results after adjusting for premorbid IQ and psychopathological symptoms measured with the PANSS. Comparison of various aspects of executive function between the two groups of patients showed no differences between the two patient groups, except for planning.

Bora [14] conducted a quantitative systematic review to assess and synthesize the available evidence on deficits in DS and NDS. The meta-analysis findings suggest that both DS and NDS are associated with cognitive impairment, relative to HC, across multiple cognitive domains. Our study showed that DS patients had lower scores on verbal working memory and cognitive flexibility than HC. NDS patients had lower planning and cognitive flexibility scores than HC. Moreover, this meta-analysis [14] indicates that patients with DS are more impaired in all cognitive domains compared to NDS. Our results do not fully confirm this, because DS patients achieved similar results for planning to HC. Cohen et al. [11] also did not find significant differences in frontal or parietal abilities for DS patients. The mini-review of Tyburski et al. [17] of 16 studies indicates that DS and NDS have greater problems with regard to nonverbal flexibility, concept formation, and problem-solving, but some of the analyzed studies did not show differences, which means that there is no consistency in terms of performance deficits between the two clinical groups. However, in this study DS patients had greater problems with concept formation compared to NDS patients, but the authors did not control for premorbid IQ.

Because general intellectual ability can be associated with executive functions and working memory both in healthy adults and clinical populations [44], it is important to include IQ assessment in the comparison of higher-order cognitive processes such as executive functions between DS and NDS patients. Previous studies yielded inconclusive results: Cascella et al. [30,31,73] found no differences between DS and NDS patients in premorbid IQ, in contrast to Wang et al. [35]. One of the explanations for such inconsistencies are that the authors use different tools to measure premorbid IQ; in the first study it was the National Adult Reading Test and in the second one—an abbreviated version of the Wechsler Adult Intelligence Scale. Our results suggest that measuring premorbid IQ should include tasks that measure both verbal and non-verbal IQ, which is consistent with the suggestions proposed by Khandaker et al. [45].

In addition, it seems reasonable to control for psychopathology dimensions when comparing executive functions between DS and NDS patients. Our results suggest that DS patients have greater problems only in planning compared to NDS patients after adjusting for psychopathological symptoms measured on the PANSS. Further research is needed to shed more light on this abstruse issue. New paradigms that will allow exploration of the nature of the relationship between psychopathological symptoms and higher-order cognitive processes in deficit schizophrenia have been postulated by Harvey et al. [74]. We tested a complex regression model for relations between clinical factors, psychopathological symptoms, and different aspects of executive functions in DS and NDS patients. We showed associations in both groups between clinical factors and aspects of executive function. We found that exacerbation had an overall effect on verbal working memory and cognitive planning in DS patients. Exacerbation turned out to be an important predictor of executive functions in this group. This means that patients who scored higher in exacerbation were likely to have poorer verbal working memory and cognitive flexibility. Moreover, in NDS patients, the SEM model confirmed that only positive symptoms are a significant predictor of cognitive flexibility. That is, patients who scored higher on positive symptoms were likely to have worse cognitive flexibility.

Dibben’s meta-analysis [29] found that negative schizophrenic symptoms and disorganization, but not positive symptoms, are significantly associated with impairment on executive tests. Strong correlations between executive dysfunction and negative symptoms and disorganization have been a feature of several studies conducted on multiple hospitalized patients [75,76]. Negative symptoms and disorganization showed similar levels of correlation with IQ as with executive impairment [29]. Intellectual impairment in schizophrenia will in itself give rise to poor performance on executive tests without implying the presence of specific neuropsychological deficits [45]. The findings of Dibben’s meta-analysis [29] support that the correlation between executive impairment and schizophrenic symptoms is specific—showing distinct patterns of association with different executive tests. The results of our analysis were not consistent with Dibben’s meta-analysis. We may have obtained different results because we included compound regression and other studies did not.

This study has advantages and limitations. The strength of our study was that we compared two clinical groups using the same neurocognitive battery. We used a consistent battery of tests that reliably measures the tested functions. Applying the five-factor PANSS model in accordance with the latest research reports is a third strength of the study. The first limitation is that the group of respondents was relatively small. The small sample of patients limits the generalizability of conclusions. It would be of great scientific value to increase the number of participants in the study. The second limitation was that the proportion of females and males in each patient group was not homogeneous (there were more male DS patients). There are some findings suggesting that female hormones benefit the brain areas involved in cognitive function [77]. This may be an alternative explanation for why NDS patients performed better on cognitive tasks than DS patients. However, it is noted that sex is one of the risk factors for deficit syndrome in schizophrenia. Previous studies show that more males than females have a diagnosis of deficit schizophrenia [78]. The third limitation is that there was a small number of patients who received mixed pharmacological treatment (typical and atypical antipsychotic medication) and we did not include this factor in the statistical analysis. There are some findings suggesting that atypical medications can improve cognitive functions in schizophrenia and future studies on the cognitive functions of DS and NDS patients should include this factor as a potentially important predictor [79]. The fourth limitation was that we used only two subtests from the WAIS-R to measure indirect premorbid IQ. Some researchers recommend cautious use of abbreviated forms when it is necessary to estimate the factor index scores and many data suggest that statistically searching for a “best” short form is largely futile [52]. Thus, short forms should be selected on the basis of their efficiency at providing the required information [49]. The fifth limitation concerns the inclusion criteria for the patient groups: illness duration of ≥10 years and age of 30–50 years. We sought to compare DS and NDS patients as a more homogenous group. However, as this may (significantly) restrict our ability to generalize our results to the entire schizophrenia population, our results should be interpreted with great caution. In addition, the study did not use ecological tests, which makes it impossible to identify disorders in the context of their impact on the daily functioning of patients. Future research should investigate cognitive dimensions using more ecological tools. Our study is cross-sectional and as such cannot control potential cohort effects. Longitudinal studies have many advantages for describing differences between clinical trials because this type of study design allows the observation of changes over time in the same participants.

## 5. Conclusions

To conclude, our study showed that executive deficits are present in both DS and NDS patients. The general severity of these deficits turned out to be greater in the DS group; however, clinical variables appear to play a significant role in their occurrence. Our findings provide evidence of a significant association between deficit syndrome and more severe global cognitive impairments in schizophrenia. Severe cognitive deficits may be relatively more strongly associated with deficit syndrome. Nonetheless, the small sample of patients limits the generalizability of conclusions. Furthermore, it is important to note that NDS is a heterogeneous concept. Some patients with NDS had persistent negative symptoms that were not considered as primary.

## Figures and Tables

**Table 1:** Demographic and clinical characteristics of all participants.

	Deficit Schizophrenia Patients (DS) (*n* = 29)	Non-Deficit Schizophrenia Patients (NDS) (*n* = 44)	Healthy Controls (HC) (*n* = 39)	*F*/*χ^2^*/*t*	η^2^/*V*/*d*
Age: *M* (*SD*)	38.59 (6.17)	39.27 (7.25)	37.08 (7.94)	0.97 ^c^	0.02 ^f^
Years of education: *M* (*SD*)	12.66 (3.24) ^i *^	13.45 (2.62)	14.59 (2.62)	4.15 ^c *^	0.07 ^f^
Sex: female/male	7/22	24/20	23/16	9.25 ^d *^	0.29 ^g^
Premorbid IQ in WAIS-R-IV:					
Picture Completion: *M* (*SD*)	17.86 (7.60)/20.52 (13.35) ^b,i^ ***^, j^ *	22.50 (6.19)/29.43 (13.37) ^b, k ***^	29.62 (3.63)/47.46 (10.34) ^b^	42.98 ^c^ ***	0.44 ^f^
Vocabulary: *M* (*SD*)	33.97 (14.47) ^i^ ***^, j^ **	43.07 (10.05) ^k ***^	56.18 (6.55)	39.43 ^c^ ***	0.42 ^f^
Antipsychotic medications:					
Atypical: *n* (%)	20 (68.97)	28 (63.64)	-	2.14 ^d^	0.17 ^g^
Atypical and typical: *n* (%)	8 (27.58)	12 (27.27)	-		
Typical: *n* (%)	0 (0.00)	3 (6.82)	-		
No medications: *n* (%)	1 (3.45)	1 (2.27)	-		
Chlorpromazine equivalent (mg): *M* (*SD*)	695.86 (311.57)	642.66 (313.15)	-	0.71 ^e^	0.17 ^h^
Duration of illness: *M* (*SD*)	16.97 (5.73)	14.09 (5.16)	-	2.23 ^e^	0.53 ^h^
Exacerbation: *M* (*SD*)	5.69 (2.44)/1.64 (0.48) ^a^	6.50 (5.07)/1.65 (0.65) ^a^	-	−0.09 ^e^	−0.02 ^h^
Global functioning in GAF: *M* (*SD*)	50.93 (14.34)	58.02 (14.15)	-	−2.09 ^e^	−0.50 ^h^
Psychopathological symptoms in PANSS:					
Positive symptoms: *M* (*SD*)	7.38 (2.73)/0.53 (0.01) ^b^	8.14 (4.39)/0.53 (0.01) ^b^	-	−0.10 ^e^	−0.02 ^h^
Negative symptoms: *M* (*SD*)	22.24 (4.66)/0.59 (0.00) ^b^	13.80 (5.25)/0.58 (0.00) ^b^	-	7.39 ^e^ ***	1.52 ^h^
Disorganization: *M* (*SD*)	12.62 (3.48)/0.54 (0.00) ^b^	11.45 (4.02)/0.53 (0.00) ^b^	-	1.90 ^e^	0.46 ^h^
Affect: *M* (*SD*)	8.24 (3.45)/0.53 (0.01) ^b^	9.25 (3.56)/0.53 (0.01) ^b^	-	−1.59 ^e^	−0.38 ^h^
Resistance: *M* (*SD*)	4.34 (0.61)/0.52 (0.00) ^b^	4.91 (2.46)/0.52 (0.01) ^b^	-	−1.13 ^e^	−0.25 ^h^
Total score: *M* (*SD*)	56.83 (11.17)/0.54 (0.00) ^b^	49.43 (14.83)/0.54 (0.00) ^b^	-	3.27 ^e^ *	0.73 ^h^
Negative symptoms in BNSS:					
Total score: *M* (*SD*)	47.07 (9.28)/0.47 (0.09) ^b^	20.23 (12.78)/0.20 (0.13) ^b^	-	9.74 ^e^ ***	2.33 ^h^
Negative symptoms in SNS:					
Total score: *M* (*SD*)	22.28 (7.38)/0.75 (0.16) ^b^	9.86 (6.90)/0.43 (0.19) ^b^	-	−7.40 ^e^ ***	−1.78 ^h^

BNSS = Brief Negative Symptom Scale; GAF = Global Assessment of Functioning; PANSS = Positive and Negative Syndrome Scale; SNS = Self-evaluation of Negative Symptoms; WAIS-R-IV = Wechsler Adult Intelligence Scale Revised Fourth Edition. ^a^ Mean and standard deviation after logarithmic transformation. ^b^ Mean and standard deviation after Box-Cox transformation. ^c^ One-way analysis of variance *F* test. ^d^ Chi-squared test. ^e^ Student’s *t* test. ^f^ Eta squared effect size: small (0.01–0.059), medium (0.06–0.139), large (0.14–1.00). ^g^ Cramer’s *V* effect size: small (0.10–0.19), medium (0.20–0.59), large (0.60–1.00). ^h^ Cohen’s *d* effect size: small (0.20–0.49), medium (0.50–0.79), large (0.80 <). All *p*-values for ANOVA. ^i^ DS patients vs. HC participants. ^j^ DS patients vs. NDS patients. ^k^ NDS patients vs. HC participants. * *p* < 0.05. ** *p* < 0.01. *** *p* < 0.001. (after Holm-Bonferroni *p*-value correction for Student’s *t* test).

**Table 2:** Comparison of different aspects of executive functions between all participants, controlling years of education and premorbid IQ.

	Deficit Schizophrenia Patients (DS) (*n* = 29)	Non-Deficit Schizophrenia Patients (NDS) (*n* = 44)	Healthy Control (HC) (*n* = 39)	*F*	η^2^
LNST: Verbal working memory: *M* (*SD*)	8.69 (3.95)/10.89 (3.42) ^a, b *^	12.14 (3.50)/12.63 (2.98) ^a^	15.87 (3.26)/13.68 (3.61) ^a^	4.29 *	0.08
SSS: Visuospatial working memory: *M* (*SD*)	5.48 (2.29)/6.73 (2.09) ^a^	7.16 (2.21)/7.50 (1.83) ^a^	9.21 (1.74)/7.89 (2.21) ^a^	2.07	0.04
MS: Planning: *M* (*SD*)	11.62 (6.35)/2.13 (2.02) ^a^	13.18 (7.77)/2.09 (1.77) ^a, c *^	21.79 (5.05)/3.41 (2.14) ^a^	4.25 *	0.07
CTT: Cognitive flexibility: *M* (*SD*)	121.93 (41.51)/4.91 (0.40) ^a,b ***^	109.70 (53.53)/4.90 (0.34) ^a, c ***^	57.10 (14.37)/4.50 (0.42) ^a^	10.45 ***	0.17
BCST PPE: Concept formation: *M* (*SD*)	18.48 (10.03)/5.98 (2.62) ^a^	17.44 (11.82)/6.00 (2.29) ^a^	12.49 (5.28)/5.77 (2.77) ^a^	0.08	0.00
BCST PNPE: Problem-solving: *M* (*SD*)	15.63 (11.37)/2.88 (0.90) ^a^	15.27 (13.90)/2.88 (0.79) ^a^	8.13 (4.18)/2.51 (0.95) ^a^	1.68	0.03

BCST = Berg Card Sorting Test; CTT = Color Trail Test; LNST = Letter Number Span Test; MS = Mazes Subtest; SSS = Spatial Span Subtest; WAIS-R-IV = Wechsler Adult Intelligence Scale Revised Fourth Edition. *F* = Analysis of covariance *F* test. η^2^ = Eta squared effect size: small (0.01–0.059), medium (0.06–0.139), large (0.14–1.00). All *p*-values for ANCOVA were after co-variates controlling years of education and premorbid IQ (scores in WAIS-R-IV: Picture Completion and Vocabulary). ^a^ Mean and standard deviation after Box-Cox transformation and corrected in ANCOVA model. ^b^ DS patients vs. HC participants. ^c^ NDS patients vs. HC participants. ^*^ *p* < 0.05. ^***^ *p* < 0.001.

**Table 3:** Comparison of different aspects of executive functions between the two patient groups, controlling premorbid IQ and psychopathological symptoms.

	Deficit Schizophrenia Patients (DS) (*n* = 29)	Non-Deficit Schizophrenia Patients (NDS) (*n* = 44)	*F*	η^2^
LNST: Verbal working memory	9.60 (3.39) ^a^	11.54 (3.32) ^a^	5.26 *	0.07
SSS: Visuospatial working memory	6.09 (1.94) ^a^	6.76 (1.92) ^a^	1.85	0.03
MS: Planning	1.65 (1.78) ^a^	1.50 (1.72) ^a^	0.12	0.00
CTT: Cognitive flexibility	5.00 (0.43) ^a^	5.04 (0.40) ^a^	0.15	0.00
BCST PPE: Concept formation	6.18 (2.85) ^a^	6.35 (2.79) ^a^	0.06	0.00
BCST PNPE: Problem-solving	3.02 (0.92) ^a^	2.93 (0.86) ^a^	0.14	0.00

BCST = Berg Card Sorting Test; BNSS = Brief Negative Symptom Scale; CTT = Color Trail Test; GAF = Global Assessment of Functioning; LNST = Letter Number Span Test; MS = Mazes Subtest; PANSS = Positive and Negative Syndrome Scale; SNS = Self-evaluation of Negative Symptoms; SSS = Spatial Span Subtest; WAIS-R-IV = Wechsler Adult Intelligence Scale Revised Fourth Edition. *F* = Analysis of covariance *F* test. η^2^ = Eta squared effect size: small (0.01–0.059), medium (0.06–0.139), large (0.14–1.00). All *p*-values for ANCOVA were after co-variates controlling negative symptoms (score on PANSS, BNSS, and SNS) and premorbid IQ (score in WAIS-R-IV: Picture Completion and Vocabulary). ^a^ Mean and standard deviation after Box-Cox transformation and corrected in ANCOVA model. ^*^ *p* < 0.05.

**Table 4:** Relationship between clinical factors, psychopathological symptoms, and different aspects of executive functions in the two patient groups.

Deficit Schizophrenia Patients (DS) (*n* = 29)						
	LNST: Verbal Working Memory	SSS: Visuospatial Working Memory	MS: Planning	CTT: Cognitive Flexibility	BCST PPE: Concept Formation	BCST PNPE: Problem-solving
	*r*	*r*	*r*	*r*	*r*	*r*
Duration of illness	−0.24	−0.11	−0.02	0.18	−0.24	0.00
Exacerbation	−0.47 *	−0.09	−0.46 *	0.48 **	0.25	0.07
Global functioning in GAF	0.16	0.06	0.37 *	−0.32	−0.19	0.02
Chlorpromazine equivalent	−0.14	−0.26	−0.02	0.05	0.00	0.33
Positive symptoms in PANSS	−0.04	−0.02	−0.26	0.28	−0.15	0.16
Negative symptoms in PANSS	0.05	−0.29	0.04	0.18	0.28	−0.15
Disorganization in PANSS	0.02	−0.05	−0.20	0.21	0.12	−0.20
Affect in PANSS	0.14	−0.17	0.18	−0.03	−0.07	−0.23
Resistance in PANSS	−0.19	−0.20	−0.16	0.40 *	−0.10	0.24
Negative symptoms in BNSS	0.10	0.16	−0.16	0.16	−0.23	−0.24
Negative symptoms in SNS	−0.13	−0.11	−0.01	0.29	−0.03	0.03
Non-deficit schizophrenia patients (NDS) (*n* = 44)						
	LNST: Verbal working memory	SSS: Visuospatial working memory	MS: Planning	CTT: Cognitive flexibility	BCST PPE: Concept formation	BCST PNPE: Problem-solving
	*r*	*r*	*r*	*r*	*r*	*r*
Duration of illness	−0.05	−0.23	−0.10	−0.09	0.08	0.06
Exacerbation	−0.08	−0.25	−0.11	0.08	−0.06	0.00
Global functioning in GAF	0.16	0.02	0.23	−0.25	−0.07	0.10
Chlorpromazine equivalent	−0.03	−0.05	−0.34 *	0.18	−0.10	−0.10
Positive symptoms in PANSS	−0.29	−0.16	−0.27	0.35 *	0.10	0.07
Negative symptoms in PANSS	0.05	0.06	−0.08	0.12	0.06	−0.13
Disorganization in PANSS	−0.18	−0.18	−0.35 *	0.30	0.28	−0.13
Affect in PANSS	−0.04	−0.20	−0.10	0.09	0.06	0.03
Resistance in PANSS	−0.03	0.03	−0.21	0.12	−0.18	−0.19
Negative symptoms in BNSS	−0.23	−0.18	−0.18	0.21	0.29	−0.09
Negative symptoms in SNS	−0.23	−0.18	−0.18	0.21	0.29	−0.09

BCST = Berg Card Sorting Test; BNSS = Brief Negative Symptom Scale; CTT = Color Trail Test; GAF = Global Assessment of Functioning; LNST = Letter Number Span Test; MS = Mazes Subtest; PANSS = Positive and Negative Syndrome Scale; SNS = Self-evaluation of Negative Symptoms; SSS = Spatial Span Subtest. * *p* < 0.05. ** *p* < 0.01.

**Table 5:** Standardized regression weights for relations between clinical factors, psychopathological symptoms, and different aspects of executive functions in DS patients.

	Estimate	Lower	Upper
Exacerbation—Verbal working memory in LNST	−0.472 **	−0.694	−0.172
Exacerbation—Planning in MS	−0.369	−0.704	0.021
Exacerbation—Cognitive flexibility in CTT	0.472 **	0.177	0.684
Global functioning in GAF—Planning in MS	0.207	−0.209	0.521
Resistance in PANSS—Cognitive flexibility in CTT	0.301	−0.038	0.581

CTT = Color Trail Test; GAF = Global Assessment of Functioning; LNST = Letter Number Span Test; MS = Mazes Subtest; PANSS = Positive and Negative Syndrome Scale. ** *p* < 0.01.

**Table 6:** Standardized regression weights for relations between clinical factors, psychopathological symptoms, and different aspects of executive functions in NDS patients.

	Estimate	Lower	Upper
Chlorpromazine equivalent—Planning in MS	−0.265	−0.508	0.107
Disorganization in PANSS—Planning in MS	−0.235	−0.524	0.120
Positive symptoms in PANSS—Cognitive flexibility in CTT	0.322 *	0.036	0.553

CTT = Color Trail Test; MS = Mazes Subtest; PANSS = Positive and Negative Syndrome Scale. * *p* < 0.05.

## Data Availability

Data and materials for the experiments reported here are available from the corresponding author on reasonable request.
